# *Dictyostelium* Cell Fixation: Two Simple Tricks

**DOI:** 10.3390/mps3030047

**Published:** 2020-07-01

**Authors:** Michael Koonce, Irina Tikhonenko, Ralph Gräf

**Affiliations:** 1 Division of Translational Medicine, Wadsworth Center, NYS Department of Health, Albany, NY 12237, USA; irina.tikhonenko@health.ny.gov; 2 Department of Cell Biology, University of Potsdam, 14476 Potsdam-Golm, Germany; rgraef@uni-potsdam.de

**Keywords:** *Dictyostelium*, cell fixation, microscopy, microtubule, cytoskeleton

## Abstract

We share two simple modifications to enhance the fixation and imaging of relatively small, motile, and rounded model cells. These include cell centrifugation and the addition of trace amounts of glutaraldehyde to existing fixation methods. Though they need to be carefully considered in each context, they have been useful to our studies of the spatial relationships of the microtubule cytoskeletal system.

## 1. Introduction

The striking mobility of *Dictyostelium* makes for an interesting and impactful model system to study dynamic cellular events [1]. Directed cell movement, rapid shape changes, and internal reorganization in response to extracellular cues are frequently accentuated here over more sedentary cell types and thus provide novel access to their study and understanding. However, one of the tradeoffs in a dynamic lifestyle is that these cells can be less tightly adherent to their substrate, and thus imaging and downstream structural analyses can present challenges that must be carefully considered.

For many years, we have tinkered with fixation and staining conditions in order to image cytoskeletal arrays and GFP-tagged proteins in *D. discoideum* [2,3,4,5,6]. Likely not alone, we have been challenged with difficulties in getting cells to sufficiently flatten to facilitate imaging and to remain attached to coverslips for downstream structural processing. In this brief report, we would like to share two insights that make a difference in our work, in the hopes that they benefit others as well.

## 2. Results

### 2.1. Cell Flattening

In the early 1980′s, Fukui and colleagues developed an agar overlay procedure for *D. discoideum* that utilizes thin sheets of agarose to flatten cells and significantly improves some aspects of cellular imaging [7]. This is now a widely used method that gently restricts the experimental depth of field to around 1–3 μm. Cells under agarose can undergo division and remain viable for hours in sealed chambers. This overlay remains our preferred method for live cell imaging, enabling us to follow individual microtubule (MT) motions in a minimal number of focal planes [8,9]. However, the procedure is a bit cumbersome to use for routine cell fixation, especially for multiple coverslips. The agar sheet takes some care to set up, only covers a small portion of the coverslip, and takes slightly longer for the fixative to penetrate to the cells, and cell loss (at least in our hands) can be significant upon sheet removal for downstream cell processing. With any perturbation, there are caveats to consider when flattening cells for long periods, and thus multiple strategies can be useful to distinguish between genuine and imposed phenotypes.

**Figure 1:**
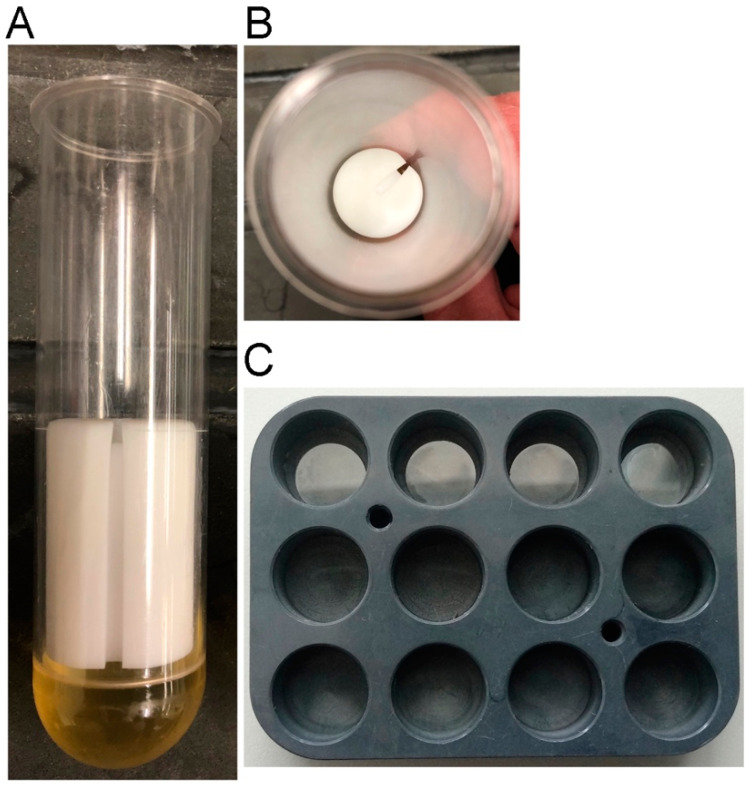
Centrifugation devices. Panel (**A**) shows a side view, panel (**B**) shows a top down view of a 50 mL Sorvall centrifugation tube fitted with an EPON base (yellow layer in **A**) and a white Delrin rod segment (2.5 cm diameter, 3.75 cm length). The notch along the side is helpful to retrieve coverslips with forceps, allowing access to the underside of the coverslip for lifting. Panel (**C**) shows a modified multiwell holder for processing up to 12 samples at a time. The top row contains 12 mm round coverslips; these can be readily removed with curved forceps.

**Figure 2:**
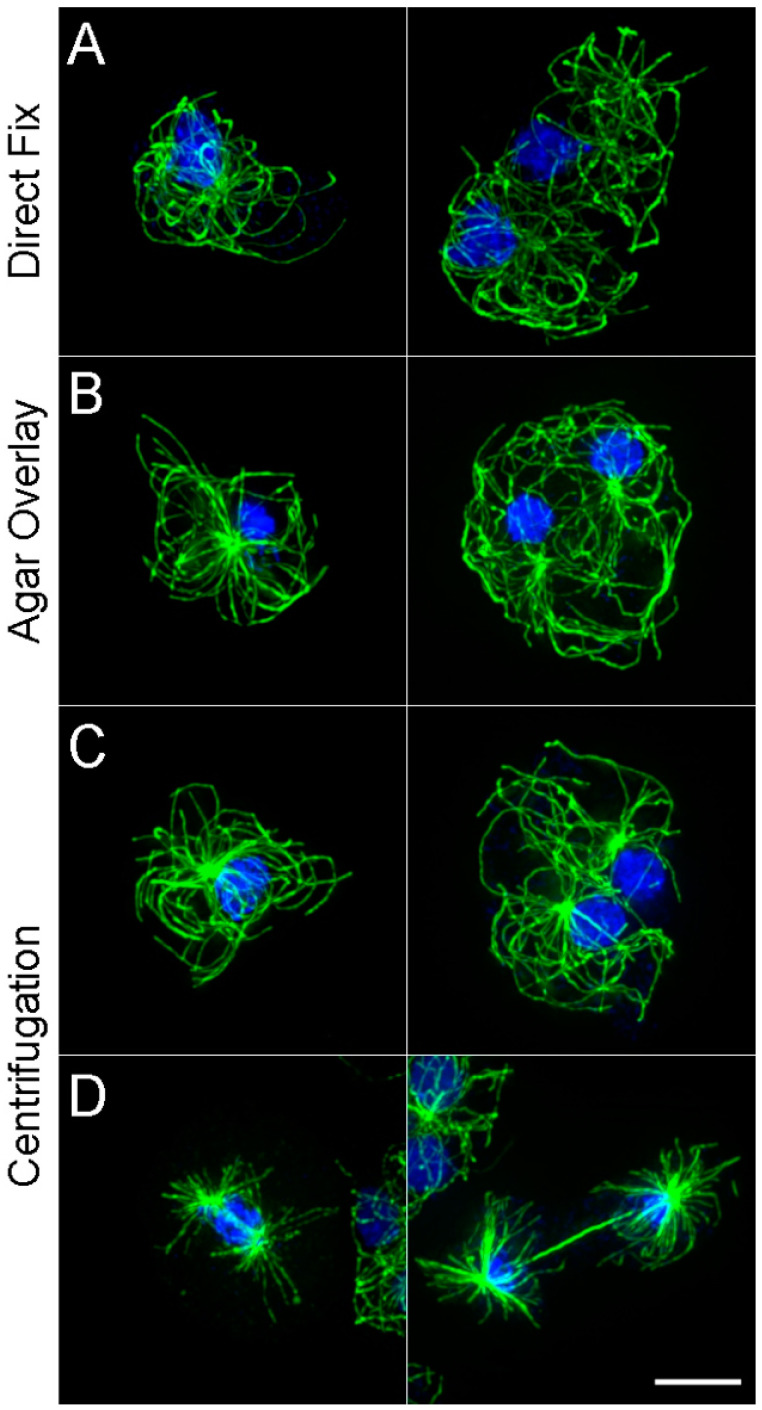
Examples of antibody-labeled microtube (MT) patterns in cells (**A**) directly added to coverslips and fixed after a 10 min period; (**B**) cells fixed after overlay with agar sheets; and (**C**,**D**) cells fixed after 5 min centrifugation onto coverslips. Panels (**A**–**C**) show interphase arrangements of mononucleated cells (left) and binucleates (right). Panel (**D**) shows mitotic cells in late anaphase (left) and late telophase (right). While the three different cell preparations look similar, it is important to note that there are typically far fewer flattened cells on the direct fix coverslips and substantially fewer cells in general on the agarose overlay coverslip. MTs are shown in green, DNA in blue, Scale bar = 5 μm.

As such an alternative, we have begun to spin cells directly onto coverslips. Five minutes at 400 g in a standard tabletop centrifuge not only promotes attachment but also cell spreading. A small volume of cell suspension is added to buffer overlying a coverslip in a centrifuge tube (Figure 1) and spun for five minutes. The coverslip is then lifted out and gently slid into a dish containing fixative. This approach eliminates the waiting period for cell settling; agarose is not required, and cells labeled with a tubulin antibody [10] appear very similar to those prepared by agar overlay (Figure 2).

The take-home message here is that the organization of the MT array in centrifuged cells appears to be very similar if not identical to what we would select as the “best” cells from either direct fix or agar overlay procedures. However, a primary benefit is that they are significantly more plentiful on the centrifuged coverslip than in the other two preparations. For example, approximately 10% of the cells on a gravity settled coverslip are sufficiently flat enough to project a useful characterization of MT arrays for the types questions that interest us. On a centrifuged coverslip, this increases to at least 60% of the population. This percentage is similar in the agar overlay preparations, but centrifuged cells extend across the entire coverslip and do not suffer detachment due to agar sheet removal.

### 2.2. Cell Attachment

**Figure 3:**
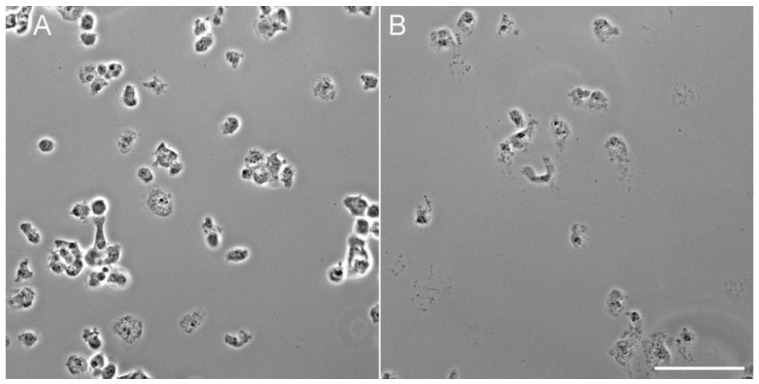
Phase contrast images of identical aliquots of *D. discoideum* cells spun onto coverslips and fixed (**A**) with or (**B**) without the addition of 0.05% glutaraldehyde and rinsed four times with buffer. Scale bar = 50 μm.

The second perhaps underappreciated impact derives from a fixation recipe that includes a small amount of glutaraldehyde (0.05% final). This concentration is not sufficient to preserve cell structure on its own, nor does it require autofluorescence quenching by sodium borohydride, but it substantially improves cell retention on the coverslip (Figure 3). Although formaldehyde alone or −20° methanol can provide structural preservation at the light microscopy (LM) level, cell loss from the coverslip is substantial at each wash or incubation step. This is particularly a problem for mitotic cells, since they are more rounded and less adherent than during interphase. The fixation recipe provided below does not interfere with GFP labels, and thus expressed tags can be imaged alongside antibody staining. Compared to the use of higher concentrations of glutaraldehyde (i.e., 0.5%) as the sole fixative, this recipe also has the advantage that it improves preservation of MTs without the often-encountered negative effects that glutaraldehyde has on antigenicity for antibodies directed against other structures.

## 3. Discussion

As with any cell perturbation, centrifugation may not universally be a good idea and needs to be approached with caution. However, side-by-side comparisons of the MT array (interphase or mitotic cells) prepared by agar overlay or centrifugation are indistinguishable, and thus in our case, we feel the method affords a useful strategy for our imaging.

An important benefit is that this method can be applied to cells that do not readily attach to the coverslip. For example, treatment of *D. discoideum* with latrunculin A (5 μM) results in actin filament disruption and cell rounding [11]. Centrifugation of Lat-A-treated cells onto coverslips followed by immediate fixation preserves a significant percentage of the population for downstream imaging. We have used this strategy to examine how the MT array may be integrated into the cortical actin meshwork.

Laboratories already comfortable with their staining procedures are not likely to do anything different, but for those frustrated that *D. discoideum* cells (or other cell types) are less adherent than mammalian tissue culture cells, these hints provide alternative strategies to consider.

## 4. Experimental Design

### 4.1. Centrifugation

For a limited number of samples, we used 50 mL polycarbonate Sorvall centrifuge tubes, which were roughly the same diameter as the ubiquitous 50 mL disposable screw cap tubes and could accommodate 18 mm^2^ coverslips. We partially filled a set with EPON resin (a common epoxy resin found in electron microscopy labs) to serve as a base, then inserted 3.75 cm long Delrin spacers of almost the same diameter as the centrifuge tube, for the coverslip to rest on. We added a small notch in the top of the spacer to facilitate forceps removal of the coverslip. In order for the spacer alone or multiple other configurations to work, one only needs a smooth surface that will support the coverslip; remain perpendicular to the centrifugal force; and, importantly, provide the ability to reach the coverslip with a pair of forceps for retrieval.

For processing more than a few coverslips, we used custom 12-(flat) well titer plates (about half the size of a standard 24-well plate) that fit into the buckets of a swing-out rotor. Round coverslips (12 mm) were placed into the wells prior to adding cells and centrifugation. There were also commercially available 24-well 0.17 mm glass bottom plates that fit into many swing-out buckets. Using these plates, the centrifugation method may more easily be applicable for live cell imaging.

A clean coverslip was added to the tube followed by sufficient phosphate buffer to cover the coverslip by about 5 mm. Using a micropipette, ~100 μL of *D. discoideum* cells were scraped from the bottom of a nearly confluent culture dish and gently added into the buffer volume directly over the coverslip. Depending on the desired cell density, this volume of cells can be adjusted. Tubes can be centrifuged as soon as convenient after adding cells; no settling period is required. While fixing *D. discoideum* cells directly in culture media should be avoided due to auto fluorescent components in some recipes, the dilution into buffer (~1:50) and time over the brief spin period is sufficient to substantially reduce the fluorescent background.

The tubes or dishes were placed in a table top clinical centrifuge with a swinging bucket rotor and spun for five minutes (400 g setting). Coverslips were then quickly removed and placed cell side up in 35 mm petri dishes prefilled with fixative, and incubated for 15–30 min. All downstream washing and incubation steps were performed as usual for antibody or reporter labeling. The centrifugation time and force settings were not rigorously optimized; we tinkered with them, and these settings worked for our purpose.

### 4.2. D. discoideum Fixative

- 50% PHEM buffer (30 mM PIPES, 12.5 mM HEPES, pH 6.9, 4 mM EGTA, 1 mM MgCl_2_). pH 6.9
- 3.7% formaldehyde
- 0.05% glutaraldehyde
- 0.1% Triton X-100

Coverslips are acid washed by soaking in 1N HCl for one hour, followed by rinsing with milli Q H_2_O, and a brief 100% ETOH wash, then air dried in a glass petri dish.

### 4.3. Phosphate Development Buffer

- 20 mM KCL
- 2.5 mM MgCl_2_·6H_2_0
- 27 mM NaH_2_PO4·H_2_0
- 24 mM Na_2_HPO_4_
- pH 6.4 (see reference [12] for detail)

### 4.4. Light Microscopy

Imaging was performed on a DeltaVision microscope workstation using the softWoRx 2.50 imaging package. Z-series stacks were collected at 0.5 μm step intervals and deconvolved. Maximum intensity projections of the entire stack are presented here, performed using FIJI [13]. DNA is labeled with Hoechst 33342. The panels were assembled using Adobe Photoshop.
